# Early Antenatal Care: Does It Make a Difference to Outcomes of Pregnancy Associated with Syphilis? A Systematic Review and Meta-Analysis

**DOI:** 10.1371/journal.pone.0056713

**Published:** 2013-02-28

**Authors:** Sarah J. Hawkes, Gabriela B. Gomez, Nathalie Broutet

**Affiliations:** 1 University College London Institute for Global Health, University College London, London, United Kingdom; 2 Amsterdam Institute for Global Health and Development, Amsterdam, The Netherlands; 3 School of Public Health, Imperial College London, London, United Kingdom; 4 World Health Organization, Geneva, Switzerland; Charité, Campus Benjamin Franklin, Germany

## Abstract

**Objective:**

Despite an increase in the proportion of women who access antenatal care, mother-to-child transmission of syphilis continues to be a consequence of undiagnosed, untreated, or inadequately treated maternal syphilis. We reviewed evidence on the optimal timing of antenatal interventions to prevent mother-to-child transmission of syphilis and its associated adverse outcomes.

**Design:**

Systematic review and meta-analysis of published literature. English-language articles were included if they (1) reported the gestational age at which the mother was screened or tested for syphilis; (2) reported on pregnancy outcome. No publication date limits were set.

**Results:**

We identified a total of 1,199 publications, of which 84 were selected for further review and five were included. All showed a lower prevalence of any adverse outcome among women who received an intervention (to include screening and treatment) in the first and second trimesters of pregnancy compared to the third trimester. The overall odds ratio for any adverse outcome was 2.24 (95% CI 1.28, 3.93). All sub-analyses by type of outcome presented important heterogeneity between studies, except for those studies reporting an infected infant (odds ratio 2.92, 95% CI 0.66, 12.87; I^2^ = 48.2%, p = 0.165).

**Conclusions:**

Our review has shown that the timing of antenatal care interventions makes a significant difference in the risk of having an adverse outcome due to syphilis. Women who sought care in the first two trimesters of their pregnancy, and received the appropriate intervention, were more likely to have a healthy infant, compared to women screened and treated in the third trimester. Encouraging ALL pregnant women to seek care in the first two trimesters of their pregnancy should be a priority for health programmes. For interventions to be effective within these health programmes, health systems and community engagement programmes need to be strengthened to enable pregnant women to seek antenatal care early.

## Introduction

Mother-to-child transmission of syphilis is a consequence of undiagnosed, untreated, or inadequately treated maternal syphilis, and can result in a number of adverse pregnancy outcomes (APOs): late foetal loss, stillbirth, low birth weight, neonatal death, or an infant with reactive serology and clinical symptoms and signs (classically called “congenital syphilis”) [1]. The likelihood of transmission (and hence the risk of adverse outcome) will vary according to both the stage of syphilis infection in the mother (primary, secondary or latent) [2], and the timing of any intervention delivered during the pregnancy [3], [4].

It has been estimated that untreated syphilis in pregnancy can directly cause adverse outcomes in around 50% of cases [5]. In comparison, HIV, if untreated, will result in in-utero transmission around 20% of the time [6], with additional transmission at the time of delivery or during breast-feeding [7], [8]. The extremely high rate of adverse outcomes seen in syphilis is probably due to direct damage caused by the spirochete (*Treponema Pallidum*) to both the placenta (microvascular proliferation and inflammation), and the umbilical cord – both of which will compromise foetal growth and viability [9].

WHO estimates that 1.3 million pregnant women annually have active syphilis infection, resulting in a substantial burden of preventable morbidity and mortality including over 200,000 stillbirths and foetal losses and over 90,000 neonatal deaths [10]. A recent systematic review of interventions to screen for syphilis among pregnant women concluded that a package of [feasible and cost-effective] interventions delivered through antenatal care services could “reduce the syphilis-attributable incidence of stillbirth and perinatal death by 50%” [11]. The intervention package included uptake of early antenatal care, decentralisation of interventions, improved clinical management, and health systems strengthening.

Access to antenatal care is one of the major achievements of maternal and child health programmes in recent years. An agreed indicator of progress towards the Millennium Development Goals (Goal 5– reproductive and maternal health), antenatal care coverage has seen marked improvement in low- and middle-income settings. The percentage of women who attend for care at least once during pregnancy has risen from 64% in 1990 to 81% in 2009 [12]. However, despite an increase in the proportion of women who access antenatal care, we still know very little about the quality of the care they receive. Moreover, we have scant information on whether pregnant women are seeking care early enough in pregnancy for antenatal interventions to have an impact, or, indeed, what is the optimal timing for many of the interventions recommended during antenatal care. The purpose of this review is to look at the evidence on the optimal timing of syphilis screening and treatment to prevent mother-to-child transmission of syphilis and its associated adverse outcomes.

## Methods

This systematic review adhered to the PRISMA guidelines for reporting of systematic reviews [13] (Text S1: PRISMA checklist).

### Search Strategy

Our study question was formulated as “Is there an increased risk of adverse outcomes associated with screening and treating women for syphilis in the third trimester compared with screening and treating in the first or second trimester?” We searched PubMed for articles with no date limitations using a search strategy combining the MESH terms: “syphilis” and “screening” and “pregnancy”. When relevant articles were located and reviewed, we searched their reference lists for additional articles. We also sought expert opinion on any additional articles which may fit the search criteria for the review.

Articles were included in the systematic review if they were publications in peer-reviewed journals, or conference papers of primary research. Full text articles were assessed and only included if they contained the following information: (1) they reported the gestational age at which the mother was screened or tested for syphilis; (2) they reported on pregnancy outcome. We considered experimental and observational designs, including prospective and retrospective cohort as well as cross-sectional studies. That the search was limited to published articles was designed to avoid poor quality studies. However, this might have led to some publication bias. Abstract and initial full-text reviews were undertaken by one reviewer (GG). Two authors (GG and SH) then reviewed full text articles and reached consensus decisions on which articles should be included in the systematic review and meta-analysis.

### Data Analysis

Data were prepared and analysed using Stata/SE 11.0 (STATA Corporation, TX, USA). Information extracted included study characteristics such as geographical location, sample size, syphilis testing, treatment available, and outcome prevalence. We calculated the crude prevalence estimate and standard error for two groups: women being tested or treated before the third trimester (i.e. at any point of the pregnancy until week 28) and those being tested or treated during the third trimester (from week 28 until delivery) [14], [15]. Effect sizes were summarized as odds ratios (ORs) and associated 95% confidence intervals (95%CI). An OR greater than one suggests that access to ANC during the third trimester increases the risk of having an adverse pregnancy outcome. We performed a random effect meta-analysis to obtain pooled estimates of ORs. Both I^2^ statistic and p values were used to assess heterogeneity between studies. We explored possible sources of heterogeneity due to characteristics of both recording of the outcome and treatment in sub-group meta-analyses [14], [15].

## Results

**Figure 1:**
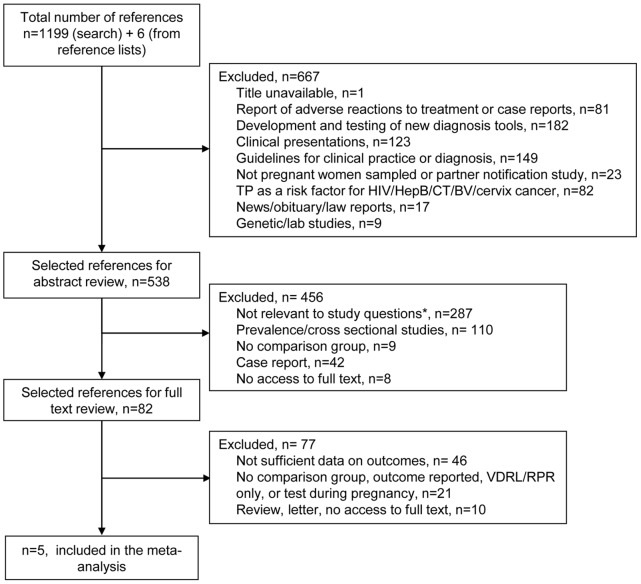
Flowchart of study selection. * includes: policy commentaries, policy analysis, clinical guidelines, best practice, commentaries/news/editorials, service audit and review, reporting analysis, patient satisfaction study, HIV+ samples, healthcare professional attitudes and practices surveys.

The initial search, conducted in November 2010, identified 1,190 articles. This search was then updated in May 2011 identifying a total of 1,199 publications. After excluding articles on the basis of not meeting the inclusion criteria noted above, the full abstracts of 538 papers were read by one reviewer and four articles were excluded because the full text was in Chinese, Hebrew, Russian, or Slovak [16], [17], [18], [19]. Of those 82 articles selected for full-text review, an additional 77 were excluded (on the decision of two reviewers) since they did not meet the inclusion criteria. Finally, five articles [20], [21], [22], [23], [24] were included (see Figure 1) and data extracted from full text articles are shown in Table 1.

**Table 1:** Study characteristics.

Reference	Country	Study type	Sample size	Syphilis diagnosis, treatment regime, and mean gestational age at treatment
**Ingraham 1951 ** [**24**]	USA	Treatment trial	663	Kahn test; aqueous penicillin by frequent injection (2.4 MU); 24 weeks*.
**Alexander 1999 ** [**23**]	USA	Prospective cohort	340	RPR or VDRL with TPHA confirmation; single dose of benzathine penicillin G (2.4 MU) intramuscular; 32 weeks.
**Watson-Jones 2002 ** [**21**]	Tanzania	Prospective cohort	382	RPR with confirmation TPHA or FTA-Abs; single dose of benzathine penicillin G (2.4 MU) intramuscular; mean gestational age at treatment: 25.3 weeks (SD 5.8; range 6.4–40.9).
**Carles 2008 ** [**20**]	Guyana	Retrospective cohort	85	VDRL or TPHA with confirmation FTA IgM; two or three doses benzathine penicillin G (2.4 MU) intramuscular; 24 weeks (range 10–40).
**Zhu 2010 ** [**22**]	China	Prospective cohort	1,469	RPR or TPPA with confirmation TPHA; for primary, secondary, early latent syphilis, benzathine penicillin G (4.8 MU) intramuscularly in two doses (9.6 MU in total) weekly. For late latent syphilis, benzathine penicillin G (2.4 MU) intramuscularly in three doses (7.2 MU in total) weekly; 25 weeks*.

RPR, rapid plasma regain test; VDRL, venereal disease research laboratory test; TPHA, *Treponema pallidum* haemagglutination testing; TPPA, *Treponema pallidum* particle agglutination assay; FTA-Abs/FTA IgM, fluorescent treponemal antibody absorbed test; MU, million units; SD: standard deviation; n, number of patients presenting the outcome. All adverse pregnancy outcomes (APOs) included: low birth weight, stillbirth, and preterm birth for Watson-Jones 2002; low birth weight, preterm birth, intrauterine death for Carles 2008; congenital syphilis, foetal death, and neonatal death for Zhu 2010.

* Estimate value from reported distribution.

Of the five full-text articles included in the meta-analysis, three studies were located in the Americas (two in the United States of America [23], [24]and one in Guyana [20]), one in Asia (China [22]), and one in Africa (Tanzania [21]). Overall, almost 3,000 pregnancies were followed and outcomes recorded (n = 2,939), and studies were conducted almost 60 years apart (papers published from 1951 to 2010). Ingraham et al and Alexander et al reported events of “congenital syphilis” only, defined as a live born child presenting signs and symptoms of syphilis infection [23], [24]. Watson-Jones et al data were included for all adverse outcomes including “prematurity” (as defined by an ultrasound/Dubovitz scale) and “stillbirth” [21]. Carles et al and Zhu et al combined the results into a single “all adverse outcomes” group [20] [22]. The definitions of cut-off points for the two groups (treatment during the first and second trimester and during the third trimester) varied across the studies. One study took a cut-off point of 28 weeks to compare women receiving care before or after this date [22]. Two studies compared women who received their first antenatal care visit and treatment before or during week 31, with women who visited/were treated after week 31 [23], [24], whilst for another study the cut-off point was week 30 [21]. The fifth study compared women receiving screening and treatment as late as 30 days before delivery with those treated earlier in their pregnancy [20].

**Figure 2:**
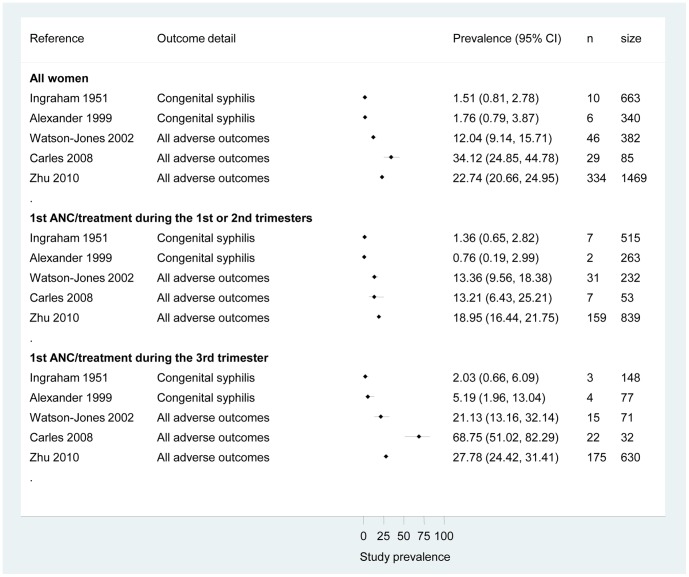
Prevalence of adverse pregnancy outcomes among all syphilis-positive women and by subgroup (tested or treated before the third trimester or during the third trimester). ANC, ante-natal care. All adverse pregnancy outcomes (APOs) included: low birth weight, stillbirth, and preterm birth for Watson-Jones 2002; low birth weight, preterm birth, intrauterine death for Carles 2008; congenital syphilis, foetal death, and neonatal death for Zhu 2010.

Prevalence of adverse outcomes is reported in Figure 2 for all studies as according to the defined subgroups based on gestational age at treatment/first antenatal care visit. Overall all studies showed a lower prevalence of any adverse outcome among women who received an intervention (including syphilis screening and treatment) in the first or second trimesters of pregnancy. Among those women who did not receive syphilis screening and treatment until the third trimester, we observed a large prevalence range depending on the outcome reported – from 2% for “classical” congenital syphilis (an infected infant) to 68% for any reported adverse outcome.

**Figure 3:**
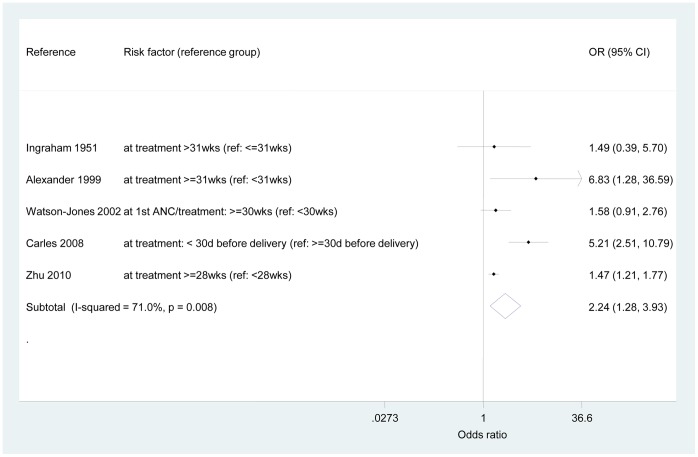
Odds ratios – adverse outcomes of women tested or treated during the third trimester compared to those women tested or treated before the third trimester (reference). Ref, reference; wks, weeks; d, days.

**Figure 4:**
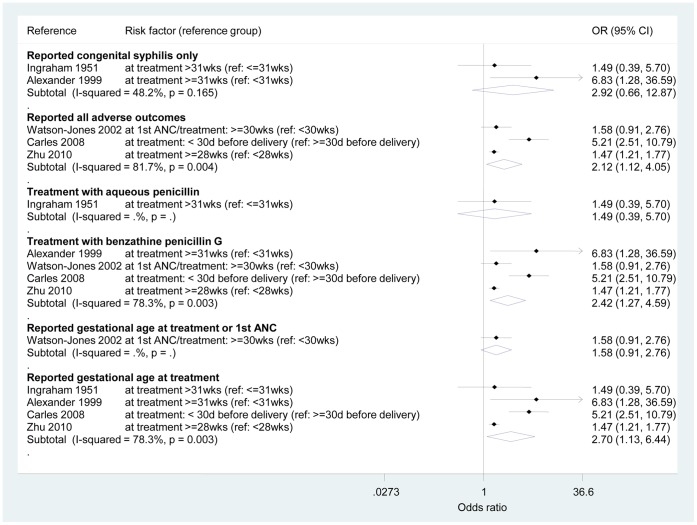
Sub-group meta-analyses. Ref, reference; wks, weeks; d, days. All adverse pregnancy outcomes (APOs) included: low birth weight, stillbirth, and preterm birth for Watson-Jones 2002; low birth weight, preterm birth, intrauterine death for Carles 2008; congenital syphilis, foetal death, and neonatal death for Zhu 2010.

The odds ratio for any adverse outcomes from being screened and treated in the third trimester compared to the first and second trimesters was 2.24 (95% CI 1.28, 3.93) – see Figure 3. However, an important amount of heterogeneity was observed (I^2^ = 71.0%, p = 0.008). To explore the sources of heterogeneity, Figure 4 shows the results of the sensitivity analyses. The OR for studies that reported only “congenital syphilis” (infected infant) events could be pooled to be 2.92 (95% CI 0.66, 12.87; I^2^ = 48.2%, p = 0.165). All other sub-analyses presented important heterogeneity between studies. However, there was a strong indication of an increased risk of adverse outcomes if women delay ANC care independently of treatment available or description of the intervention: the pooled ORs ranged from 2.42 (95% CI 1.27, 4.59; I^2^ = 78.3%, p = 0.003) for studies reporting treatment with benzathine penicillin G only compared to those reporting treatment with aqueous penicillin to 2.70 (95% CI 1.13, 6.44; I^2^ = 78.3%, p = 0.003) for studies that reported on gestational age at treatment only as opposed to gestational age at treatment or first ANC.

## Discussion

Syphilis continues to exert a high burden of disease globally [10], and in several parts of the world rates of congenital syphilis are rising [25], [26]. The extremely high rates of adverse outcomes of pregnancy associated with in-utero transmission can be substantially reduced through relatively simple and cost-effective interventions which are feasible to administer in most settings [27], include serological or whole blood screening, and treatment with penicillin for women (and their partners) found to be seropositive. Recently, WHO has called for the global elimination of mother-to-child transmission of syphilis, through commitment to and implementation of four strategic pillars – advocacy and political commitment; early access to maternal and neonatal health care; screening all pregnant women; surveillance, monitoring and evaluation [28]. Pillar 2 of the WHO global strategy explicitly mentions a goal of “increasing the percentage of pregnant women attending….facilities *early* in pregnancy” [28]. However, to date, there has been no review of the effectiveness of early interventions to prevent mother to child transmission of syphilis.

This review represents the first systematic evaluation of the impact of screening and treating women early in pregnancy compared to later in pregnancy. We have defined “early” in its broadest terms – the first and second trimesters (i.e. up to and including week 27 of the pregnancy). This was mainly done for pragmatic reasons – antenatal care is often not sought till at least the second trimester in many settings [29], therefore we assumed *a priori* that there would be insufficient data to allow us to analyse the impact of screening and treatment in the first trimester alone.

We identified five studies which compared women seeking and receiving interventions (screening and treatment for syphilis) in the first and second trimesters (reference group) compared to those women not seen until the third trimester [20], [21], [22], [23], [24]. Overall prevalence of any adverse pregnancy outcomes was higher among women seen in the third trimester compared to the reference group for all studies – resulting in an odds ratio of 2.24 (for adverse outcomes among women seen later in their pregnancies). There was an important heterogeneity observed between studies, and results might have been affected by individual study designs. According to the results of our meta-analyses by sub-groups, the main characteristic that helped explain the heterogeneity observed was the type of outcome reported.

Screening and treatment earlier in the pregnancy had an impact, in general,on the risk of all adverse outcomes (OR = 2.24 (95% CI 1.28, 3.93)), and in particular, on the risk of ‘congenital syphilis’ (i.e. an infant with evidence of infection; OR = 2.92 (95% CI 0.66, 12.87)). However, only the studies reporting on congenital syphilis events [23], [24] were statistically similar and their estimates could be confidently pooled. Among those studies reporting on all adverse outcomes, there were some methodological differences. For instance, Watson-Jones et al [21] included low birth weight, stillbirth, and preterm birth in their definition of all adverse outcomes, and presented detailed data on individual pregnancy outcomes. Zhu et al [22] included congenital syphilis (i.e. an infected infant), foetal death, and neonatal death in their definition, and included mothers with primary, secondary, and tertiary syphilis in their analysis. Both of these studies might have underestimated the rate of adverse outcomes. Meanwhile, Carles et al [20] looked at low birth weight, preterm birth, and intrauterine death in a population accessing services very late during the pregnancy or at birth.

Overall, there was an increased risk of prematurity in mothers presenting late to ANC, with an odds ratio of 2.09 (95% CI 1.09–4.00). For stillbirth, we found an odds ratio of 0.71 (95% CI 0.21–2.48; not significant), probably due to the small number of events observed (n = 15 stillbirths overall, only three in women presenting to ANC during the third trimester).

As noted, antenatal care is now accessed at least once in pregnancy by a majority of women. This is a significant public health achievement and a reflection of the highest level of global commitment to ensure that all pregnant women can access the antenatal care they need [30], which has helped to mobilise resource allocation in this area. However, the global goals and commitments for antenatal care do not, at present, include a target for ensuring that women seek care early enough in pregnancy for interventions to be most effective.

We still know little about “what works” in terms of successfully enabling women to seek and receive antenatal care early in their pregnancies. As mentioned, women face numerous obstacles to early antenatal care, including cultural barriers [31], economic barriers [31], [32], and misconceptions about the perceived and actual benefits of early antenatal care [33]. We have little empirical or trial evidence on how to increase early antenatal care attendance. A non-randomised controlled trial conducted in Zambia in the late 1980s showed that a multi-pronged intervention including behaviour change messages aimed at whole communities as well as specific groups in those communities had the effect of increasing the percentage of pregnant women who sought ANC in the first trimester from 9.4% (68/723) to 42.5% (194/457) [34]. Components of the successful intervention included provision of health information about the importance of early antenatal care to groups identified as sexually active (for example, women at family planning clinics, men and women at out-patient clinics), and to elderly community members who have a specific role as community leaders and influencers. Health education messages were developed in collaboration with local communities, and were given repeatedly. However, additional high quality (for example, randomised controlled trial) evidence is currently lacking in this area.

Our review has clearly shown that the timing of antenatal care interventions to prevent adverse outcomes due to syphilis makes a significant difference in outcome rates. Women who sought care in the first two trimesters of their pregnancy, and received the appropriate intervention, were more likely to have a healthy infant, than those who waited until the third trimester before seeking care. Such findings carry important implications for antenatal care programmes, and whether or not similar findings are observed with other antenatal care interventions (such as preventing mother to child transmission of HIV) needs further exploration.

Encouraging ALL pregnant women to seek care in the first two trimesters of their pregnancy to avoid preventable adverse outcomes should be a priority for health programmes. This is likely to be achievable, in part, with strengthening of health systems so that health workers are able to provide high quality comprehensive antenatal care for all women. Screening for syphilis and HIV during the first ANC visit is recommended. However, the timing of this visit in addition to whether these tests were performed and treatment received should be seen as indicators of high quality antenatal care. Early access to ANC will also be enhanced by community engagement programmes that work with women, their families and their communities to enable pregnant women to seek antenatal care as early as possible for interventions to be effective – the mechanisms and methods by which to achieve this deserve further attention.

## Supporting Information

Text S1 **PRISMA checklist.**
(DOCX)
